# Use of Assistive Technology for Persons with Psychosocial Disability: Systematic Review

**DOI:** 10.2196/49750

**Published:** 2023-11-15

**Authors:** Ikenna D Ebuenyi, Celina Flocks-Monaghan, Sarju S Rai, Ralph de Vries, Soumitra S Bhuyan, Jonathan Pearlman, Nev Jones

**Affiliations:** 1 Department of Rehabilitation Science and Technology University of Pittsburgh Pittsburgh, PA United States; 2 School of Population Health Royal College of Surgeons in Ireland Dublin Ireland; 3 Athena Institute VU University Amsterdam Amsterdam Netherlands; 4 Medical Library Vrije Universiteit Amsterdam Netherlands; 5 Edward J. Bloustein School of Planning and Public Policy Rutgers University New Brunswick, NJ United States; 6 School of Social Work University of Pittsburgh Pittsburgh, PA United States

**Keywords:** assistive technology, assistive products, psychosocial disability, inclusion, participation, rehabilitation, psychosocial, health policy, socioeconomic, well-being

## Abstract

**Background:**

Assistive technology (AT) refers to assistive products (AP) and associated systems and services that are relevant for function, independence, well-being, and quality of life for individuals with disabilities. There is a high unmet need for AT for persons with disabilities and this is worse for persons with cognitive and mental or psychosocial disabilities (PDs). Further, information and knowledge on AT for PDs is limited.

**Objective:**

The aim of this review was to explore the pattern of AT use among persons with PDs and its associated socioeconomic and health benefits.

**Methods:**

The review was reported according to the PRISMA (Preferred Reporting Items for Systematic Reviews and Meta-Analyses), and we conducted systematic searches in the 4 databases: PubMed, Embase.com, APA PsycInfo (Ebsco), and Web of Science (Core Collection) with the following index terms: “Assistive Technology,” “Self-Help Devices,” “Quality of Life,” “Activities of Daily Living,” “Mental Disorders.” We included only AT individuals with PDs can independently use without reliance on a provider. Identified papers were exported to EndNote (Clarivate) and we undertook a narrative synthesis of the included studies.

**Results:**

In total, 5 studies were included in the review which reported use of different AT for schizophrenia, bipolar disorder, depression and anxiety disorders. The APs described in the included studies are Palm tungsten T3 handheld computer, MOBUS, personal digital assistant, automated pill cap, weighted chain blankets, and smartphone function. All the AT products identified in the studies were found to be easily usable by individuals with PDs. The APs reported in the included studies have broad impact and influence on social function, productivity, and treatment or management. The studies were heterogeneous and were all conducted in high-income countries.

**Conclusions:**

Our study contributes to and strengthens existing evidence on the relevance of AT for PDs and its potential to support socioeconomic participation and health. Although AT has the potential to improve function and participation for individuals with PDs; this review highlights that research on the subject is limited. Further research and health policy changes are needed to improve research and AT service provision for individuals with PDs especially in low-income settings.

**Trial Registration:**

PROSPERO CRD42022343735; https://www.crd.york.ac.uk/prospero/display_record.php?RecordID=343735

## Introduction

Assistive technology (AT) is a generic term used to refer to assistive products (AP) and associated systems and services that are relevant for maintaining or improving an individual’s functioning, independence, well-being, and quality of life [1,2]. The 2022 global report on AT suggests that AT is relevant for inclusion and its use extends beyond persons with disabilities to include populations of persons with chronic conditions and also persons without disabilities [1]. Globally, about 2.5 billion are in need of AT, and this is expected to increase to over 3.5 billion people by 2050 [1].

This high unmet need for AT cuts across all types of disabilities but is often worse for AP related to mental and cognitive disabilities. The predominant examples of AT use and provision are often for physical disabilities, such as walking devices or hearing aids. However, individuals with psychosocial disabilities (PDs) need AT and would benefit from AT [1]. PDs are those disabilities that may arise on account of mental health conditions, and which hinder those individuals from leading independent and functional lives [3,4]. Functional capacity, which refers to the ability to perform tasks and activities necessary or desirable in life is an essential component of independent living and diagnostic criteria for mental health and neuropsychological disorders [5]. The World Health Organization estimates that a quarter of the world’s population will have some form of mental health condition in their lifetime [6]. In the United States alone, nearly 1 in 5 adults live with a mental illness (57.8 million in 2021) which substantially interferes with or limits their daily life activities [7]. The 2022 global report on AT enumerates the benefits on APs for mental health to include “person-centeredness, convenience, ease of accessibility and different modes of accessibility, increased coverage and availability of services, cost effectiveness” [1,8].

Therefore, the usage of AT for individuals with PDs is critical. Ringland et al [9] highlights the need to understand mental illness as a PD and the importance of provision of AT to improve function. AT may support activities such as scheduling assistance, task management, calming and comforting, mindfulness, and distraction [8,10,11]. AT products that support mental function include items such as watches, electronic calendars, custom-made PDA (personal digital assistant), weighted or ball blankets, cell phones (using special mobile apps). Emphasizing patient independence, AT provides tools for self-management of mood and behavior tracking, monitoring of sleep and diet, symptom tracking, self-awareness of breathing rate, and self-measurement of pulse [8]. For example, in patients with schizophrenia, AT can provide blocking or managing auditory hallucinations with music or audio files, medication management, tools for connecting with community, monitoring of symptoms, and identification of coping strategies [12,13]. A report by the Nordic Center for Welfare and Social issues suggests that AT for persons with mental health problems have economic benefits such as increased ability for employment, reduced need for care, and reduced use of health services [8]. The report underscores the importance of AT for PDs and describes the usefulness of AT such as a visual countdown timer for improved concentration for persons with manic depression; PDA in control over tasks for individuals with schizophrenia and ball blanket for relief of anxiety [8].

Despite the known improvements AT can provide for persons with PDs, the research on use of AT for mental health is limited [1,14]. The United Nations Convention on Rights of persons with disabilities recommends equitable use and provision of AT for persons with disabilities [15]. Research on use and provision of AT for individuals with PDs is relevant for socioeconomic, independence and well-being of affected individuals. The aim of this review is to respond to this need by exploring the use of AT for persons with PDs and associated socioeconomic and health benefits. In this study, we focused specifically on what AT individuals with PDs can use independently which are not provider dependent. Studies endorse the importance of user centered AT and its relevance in promoting use of AT and their independence.

## Methods

### Study Design

This review is reported according to the PRISMA (Preferred Reporting Items for Systematic Reviews and Meta-Analyses) [16]. The protocol for the review was registered in a PROSPERO (prospective register for systematic reviews) [17].

### Search Strategy

To identify the relevant publications, we conducted systematic searches in the bibliographic databases PubMed, Embase.com, APA PsycInfo (Ebsco) and Web of Science (Core Collection) from inception up to December 20, 2022, in collaboration with a medical information specialist. The following terms were used (including synonyms and closely related words) as index terms or free-text words: “Assistive Technology,” “Self-Help Devices,” “Quality of Life,” “Activities of Daily Living,” and “Mental Disorders.”

The references of the identified papers were searched for relevant publications. Only studies in the English language were accepted. Duplicate papers were excluded by a medical information specialist using EndNote (version 20.0.1; Clarivate), following the Amsterdam Efficient Deduplication–method [18] and the Bramer-method [19]. The full search strategies for all databases can be found in Multimedia Appendix 1.

### Data Collection

This review includes studies on the use of AT for mental health problems. Specifically, populations diagnosed with anxiety, depression, bipolar disorders, and schizophrenia, in adults older than 18 years. For the purpose of this review, our focus was on PDs; hence, studies on cognitive disabilities and intellectual disabilities were excluded [20,21]. Although AP includes software, mobile-health, eHealth programs, and other internet-based services which rely on provider-led platforms, in this review we sought to include only hardware-based AP that may be independently used by the individual [1].

**Figure 1:**
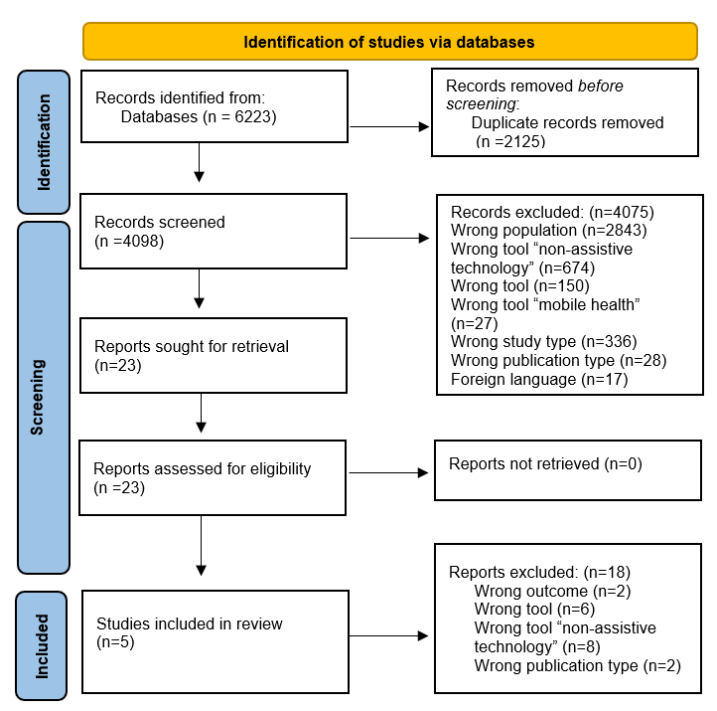
PRISMA (Preferred Reporting Items for Systematic Reviews and Meta-Analyses) flowchart of the study selection process.

All relevant papers relating to the usage of AT for PDs until December 2022 were captured. Identified papers were exported to EndNote. The literature search generated a total of 6223 references: 1878 in PubMed, 2844 in Embase.com, 570 in APA PsycInfo, and 931 in Web of Science. After removing duplicates of references that were selected from more than 1 database, 4098 references remained. Further, 2 reviewers CFM and IDE independently screened all potentially relevant titles and abstracts for eligibility. If necessary, the full-text paper was checked for the predefined eligibility criteria (Textbox 1). Differences in judgement were resolved through a consensus procedure. Studies were included if they met the following criteria. After screening, 23 papers were selected for full-text screening; out of which five were selected for the final review The flowchart of the search and selection process is presented in Figure 1.

**Eligibility criteria for study selection.**

**Inclusion criteria**

- Studies on the utilization of assistive technology for persons older than 18 years who have been diagnosed with or are suspected of having a psychosocial disability
- Assistive technology or assistive products hardware that are under the control of the user
- Studies regarding disability associated with anxiety, depression, bipolar disorders, and schizophrenia
- Outcomes on function, treatment, and socioeconomic participation
- English language

**Exclusion criteria**

- Studies focusing on the use of assistive technology by individuals under the age of 18 years or not written in English will be excluded
- Assistive technology software
- Studies regarding autism and cognitive disabilities such as dementia or intellectual disabilities
- Other outcomes unrelated to outcome of interest
- Non-English language studies

### Data Synthesis

To synthesize the collected evidence, an extraction table was created. The following items were included: the authors, the aims, study design, country, PD population (ie, age, type of PD, setting, and outcome), APs and pattern of use, analysis, outcome (socioeconomic participation and health), and quality assessment. Quality assessment was conducted by CFM and reviewed together with IDE using the Mixed Methods Critical Appraisal Tool [22]. The Mixed Methods Critical Appraisal Tool is a systematic appraisal tool where 2 independent reviewers score the quality of the included studies [22]. The extraction was conducted by CFM and reviewed together with IDE. A summary of the data extraction is presented in Multimedia Appendix 2. Due to the limited and heterogeneous nature of the studies included in the review, a narrative synthesis was undertaken to highlight the use of AT for PDs.

## Results

### Study Characteristics

The characteristics of the 5 studies [23-27] included in the analysis are presented in Multimedia Appendices 2 and 3. The studies were heterogenous in their objective, country of origin, study design, AT type, and study population and were published between 2008 and 2021. In total, 2 studies were undertaken in the United States [23,25], and the other 3 were each in France, Sweden, and Italy [24,26,27]. Four of the studies were quantitative with three using experimental and randomized control trial design [24,26,27], one adopting a prospective design [25] while 1 study was a single patient case study report [23]. Of these 5 studies, two focused on AP for schizophrenia [23,24], one was specific for bipolar disorder [25], one was for a mixed group of depression, anxiety, and bipolar disorder patients [26], and one was for a combined group of schizophrenia and depression patients [27]. The quality of the included studies ranged from moderate to strong. Further, 3 studies were of moderate quality [23,24,27] while 2 were of strong quality [25,26]. In the next sections, we will describe APs reported in included studies and their *pattern of use* for individuals with PDs and the outcomes reported in the studies that supports their *socioeconomic participation or health.*

### APs and Pattern of Use

The analysis found 2 broad categories of AP; 3 studies focused on devices in the form of digital devices [23,24,27] and 2 studies reported devices with atypical digital element [25,26]. All digital devices could be used independently by the patient, although some needed preprogramming from the study researchers.

In total, 2 studies used versions of PDAs [23,24]. The Palm digital computer was used in the Kimhy and Corcoran [23] study as a means of complementary treatment with provider led cognitive behavioral therapy. The Palm computer was employed for the case report for a patient with schizophrenia [23]. The device prompted the user to input information regarding changes in thoughts, mood, behavior, and social contexts throughout the day [23]. The utility of a PDA device was also seen in the Sablier et al [24] study with schizophrenia patients, through the MOBUS device. The MOBUS device prompted the user to record symptoms of their condition when conducting activities of daily living (ADL) [24]. The device allowed the user to track their symptoms of “distress,” “tiredness,” and “voices,” along with a scale of the level of which they were experiencing the symptom [24].

The usage of a smartphone as a form of AP was tested in the study by Resta et al [27]. While the default for smartphone usage as a means of AT is to rely on specialty developed apps, this study highlighted the utility of the phone itself. The study used Samsung galaxy phones equipped with alarms which were set up to provide verbal reminders at the time an activity was due and then provided verbal instructions for the single activity steps [27].

In total, 2 studies focused on atypical digital devices, one testing the effectiveness of a pill cap monitor [25] and another treating insomnia with a weighted blanket [26]. The study regarding the pill cap monitor recorded instances of the bottle opening and stored it in memory chip, tracking patients’ presumed dosing episodes [25]. This small chip kept track of the number of openings per day for an 8-day period, data which could be viewed by providers [25]. For the user, the pill cap served as an external marker for keeping track of time of last dosing. For instance, 1 user was meant to take a medication every 8 hours and the monitor kept them informed of the time since last opening and presumed first daily dosage [25].

The weighted chain blanket was to be used nightly for combatting difficulties in falling asleep, problems staying asleep, and the subsequent daytime symptoms connected to sleeping issues in individuals with major depressive disorder, bipolar disorder, and generalized anxiety disorder [26]. The physical weight of the chain blanket was determined to have a calming sensory effect through deep pressure stimulation [26].

### Outcomes and Related Socioeconomic Benefits

The studies analyzed reported influences of the different AP on the individuals’ daily functioning and socioeconomic benefits. These outcomes may be broadly classified into social function, productivity, and treatment or management.

### Social Function

The APs impact on participants' social settings and day-to-day activities was a prominent outcome observed in multiple studies. These studies consistently revealed notable improvements in social capabilities and overall functionality resulting from the use of these devices. For instance, the study conducted by Kimhy and Corcoran [23] emphasized how participants experienced enhanced recognition of dysfunctional thoughts with the aid of the PDA tool. Moreover, the PDA device served as a valuable confidence-building tool, enabling users to express themselves more productively and clearly [23]. Similarly, in the Sablier et al [24] study, the utilization of the PDA device was associated with increased ambition to engage in new activities and greater willingness to socialize. Notably, the use of weighted blankets to combat insomnia yielded an additional positive outcome by reducing daytime fatigue, which facilitated behavioral activation—an important factor for individuals with depression [26].

### Productivity

One study’s findings described the use of the AT for improving participants’ ability to complete coursework tasks [23]. Further, the AT aided in an increased interest in activities with a greater sense of competency toward them [23]. The Resta et al [27] study using the smartphone functions, reported a significant increase in the number of activity steps correctly performed when supported by the devices’ prompting.

The secondary impacts of the utilization of the weighted blankets were reported as increased rates of activity, and patients were able to sustain daily activity for longer [26]. This was attributed to increased sleep maintenance, and decreased reports of resting periods needed during daytime activities, as well as reduced symptoms of fatigue [26].

### Treatment or Management

A key outcome for 3 of the studies analyzed was the improvement in independent treatment management [23,25,26]. In the study by Ekholm et al [26] the targeting of insomnia led to an antidepressive effect on the participants. In this study, depressive and anxiety symptoms decreased significantly for participants allocated the weighted blanket which was attributed to the cyclical correlation between insomnia and depression [26].

Reduction in symptoms of anxiety, guilt, and depression was also reported in the patient in the study by Kimhy and Corcoran [23]. The utilization of the PDA device allowed the user to develop feelings of hopefulness and ambition toward their future [23]. Further, the utility of the pill-cap monitors in the Sajatovic et al [25] study showed clear improvement in adherence to medication and treatment.

## Discussion

### Summary of Findings

This review highlights the use of a variety of AT for PDs including schizophrenia, bipolar disorder, depression, and anxiety disorders. However, it also reveals a dearth of empirical studies on AT that individuals with PDs can use independently. This finding has several implications and may be subject to several interpretations. First, the selected 5 studies indicated relevance of AT for increased productivity (eg, in school work), increased motivation to try new activities and social function, improved medication adherence, improved sleep, and ADLs. These benefits demonstrate the need to prioritize AT for use by individuals with PDs. It also aligns with the report of the Nordic Center for Welfare and Social on AT use for mental health and the benefits for social function [8].

On the other hand, the limited nature of studies on the subject may imply an overarching lack of awareness of the benefits AT can have on the lives of those with these specific PDs. The lack of understanding about mental illness and its recognition as a disability is not new. This may explain the study by Ringland et al [9] that attempts to make a case for understanding mental ill-health as a PD and the importance of AT in this regard. The critical need to prioritize AT for mental health underscores its coverage in the 2022 AT report and call to prioritize coverage of AT based on peoples’ needs [1]. Second, few identified studies may imply that the AT needs of individuals with PDs are not met especially in low- and middle-income countries. This evidence is based on the fact that all the studies included in this review are from high-income countries. Despite the limited number of studies recorded in this review, the included relevant literature provides important findings on AT types for PD and their critical user impact.

This review reiterates the relevance of different AT types for schizophrenia, bipolar disorder, depression, and anxiety disorder [23-27]. These findings are corroborated by previous studies which have reported AT use in different mental health problems [8,10,11,17,28]. The different first groups of AT reported in this review were digital in nature in the form of PDA [23,24] and the smartphone [27]. The rise in use of digital mental health apps and recognition of their importance has been previously reported [13]. The majority of these phone apps are outside the control of the individual or provider-controlled [13,29]. In this study, we have focused on AT within the control of the users. The potential for use of an AT in combination with machine learning to predict panic attacks has also been reported [30]. The second group of APs found in our review were not typically digital. The pill cap monitor and the weighted chain blankets reported in 2 different studies were reported as useful for individuals with different forms of PDs [25,26].

Our review underscores the relevance of different AP for improved social function, productivity, and treatment for individuals with PDs. While the PDA in the study by Kimhy and Corcoran [23] helped in the recognition of dysfunctional thoughts, the one by Sablier et al [24] increased the interest to try new activities. These reported outcomes are essential for improved function and participation for individuals with PDs and ought to be available for those who need them similarly, the weighted blanket was reported to increase daytime activity levels and reduce daytime symptoms of fatigue, anxiety, and depression [26]. The potential of PDs to reduce energy levels and reduction in social participation has been previously reported [5]. The reported roles of the AP to help with these functions imply opportunities to improve the functional capacity of affected individuals and ADL.

Similarly, this review noted the impact of the AP on productivity. The PDA in the case report was reported to increase the ability to complete tasks [23] while the smartphone in Resta et al [27] increased the number of activity steps correctly performed. Further, the weighted blankets were reported which were reported to increase activities and may help boost an individual’s productivity [26]. These findings have implications for use in individuals with low energy on account of PDs and may be adapted for use to help improve ADL.

Further, three of the studies included in the review reported improvement in the independent treatment management [23,25,26] for individuals with PDs. The implication of these findings is that the PDA may be used to improve the feeling of hopelessness [23] which is a cardinal feature in depression [5]. The pill cap’s usefulness in improving treatment adherence may be adapted for individuals with other PDs having difficulties adhering to their medications [25]. The findings in these studies indicate that with adequate support, individuals with PDs can lead independent and productive lives which may improve their overall health outcomes [3]. The evidence suggests that the APs may offer reduced need for caregiver assistance and health care services. The potential of the weighted blankets to reduce depressive and anxiety symptoms in participants allocated the weighted blanket implies opportunities to use them for individuals who indicated a need for them.

This review highlights the lack of available options for individuals who may not have access to AP. Only about 10% of individuals in need of AT have access to them [13]. Of note, this estimate refers more to individuals with physical disabilities than those with PDs. This is true in low- and middle-income countries which is not represented in the countries included in the study. It is also pertinent to point out that electronic or digital APs may not be suitable or accessible for all patients, particularly the elderly or those with comorbidities such as learning disabilities or impaired. It is essential that interventions and policy actions on use and provision for APs for PDs conceives options to include and cater for the needs of such individuals.

This review has some limitations. First, our eligibility criteria meant that we only included AT products that were under the control of the user. We opted for this as it would have otherwise included several digital mental health, smartphone, and app-based tools which are sometimes provider-dependent and outside the control of the user. Through the study screening process, the reliance on smartphone apps as the “go-to” tool for individuals with PDs was prominent. APs that users can use independently are easier to use. Further, ethical and safety concerns of provider-controlled digital and AT products for mental health have been previously reported [13,29]. Furthermore, the inclusion of only English studies limited the scope of the review.

### Conclusions

This review revealed reported use of different AT for PDs including schizophrenia, bipolar disorder, depression, and anxiety disorders, that showed benefits in increasing productivity (eg, in school work), motivation to try new activities and social function, improving medication adherence, sleep, and ADLs. Yet, we noted that these studies were limited in number and scope, and there were no studies from a low-and-middle country. This review indicated a pressing need for more empirical research focusing on the utilization, user experience, and evaluation of ATs tailored to individuals with PDs, especially from low-and-middle income countries. Such studies are essential for informing evidence-based policies and practices, ultimately leading to improved functioning, enhanced participation, and the promotion of inclusive environments for individuals living with PDs.

## Appendix

Multimedia Appendix 1 Search strategy for databases.

Multimedia Appendix 2 Study characteristics, objectives, and assistive product use and outcomes.

Multimedia Appendix 3 Assistive technology for psychosocial disability.

Multimedia Appendix 4 PRISMA Checklist.

## Abbreviations

ADL: activity of daily living
AP: assistive product
AT: assistive technology
PD: psychosocial disability
PDA: personal digital assistant
PRISMA: Preferred Reporting Items for Systematic Reviews and Meta-Analyses
PROSPERO: prospective register for systematic reviews

## Data Availability

The data used in this study are available from the corresponding author upon reasonable request.
