# Supplementary material for: Use of Assistive Technology for Persons with Psychosocial Disability: Systematic Review
Source: JMIR Rehabil Assist Technol. 2023 Nov 15;10:e49750. doi: 10.2196/49750 (PMC10687692; doi:10.2196/49750)
Supplement: Multimedia Appendix 1 [file rehab_v10i1e49750_app1.docx]

**Multimedia Appendix 1.** Search strategy for databases.

**PubMed Session Results (20 Dec 2022)**

| Search | Query | Items found |
| --- | --- | --- |
| #5 | **#4 NOT (("Child"[Mesh] OR "Infant"[Mesh] OR child*[tiab] OR schoolchild*[tiab] OR infant*[tiab] OR girl*[tiab] OR boy[tiab] OR boys[tiab] OR boyhood[tiab] OR youth*[tiab] OR pediatr*[tiab] OR paediatr*[tiab] OR puber*[tiab]) NOT ("Adult"[Mesh] OR "Adolescent"[Mesh] OR adult*[tiab] OR adolescen*[tiab] OR man[tiab] OR men[tiab] OR woman[tiab] OR women[tiab] OR teen[tiab] OR teens[tiab] OR teenager*[tiab]))** | 1,878 |
| #4 | **#1 AND #2 AND #3** | 1,965 |
| #3 | **"Mental Disorders"[Mesh] OR "Mentally Ill Persons"[Mesh] OR "Persons with Mental Disabilities"[Mesh] OR "severe mental"[tiab] OR psychosis[tiab] OR psychoses[tiab] OR psychotic[tiab] OR schizo*[tiab] OR bipolar*[tiab] OR "mental disab*"[tiab] OR "mentally disab*"[tiab] OR "psychiatric disab*"[tiab] OR "psychosocial disab*"[tiab] OR "psycho-social disab*"[tiab] OR "major depress*"[tiab] OR "anxiet*"[tiab] OR "depressive"[tiab]** | 1,708,343 |
| #2 | **"Quality of Life"[Mesh] OR "quality of life"[tiab] OR "life qualit*"[tiab] OR "living qualit*"[tiab] OR "quality of living"[tiab] OR "Activities of Daily Living"[Mesh] OR "activities of daily living"[tiab] OR "activity of daily living"[tiab] OR "activities of daily life"[tiab] OR "activity of daily life"[tiab] OR "daily living activit*"[tiab] OR "daily life activit*"[tiab] OR "daily time"[tiab] OR "time management"[tiab] OR "adl"[tiab] OR "qol"[tiab] OR "hrql"[tiab] OR "hrqol"[tiab] OR participation[tiab] OR "social engagement"[tiab] OR "social relation*"[tiab] OR "social inclusion"[tiab] OR "social accept*"[tiab] OR "independent living*"[tiab] OR "living independent*"[tiab] OR "everyday function*"[tiab] OR "daily function*"[tiab] OR "activities of everyday"[tiab] OR "everyday liv*"[tiab] OR "everyday lif*"[tiab] OR "daily activ*"[tiab] OR "daily task*"[tiab]** | 738,659 |
| #1 | **"Self-Help Devices"[Mesh:NoExp] OR "Communication Aids for Disabled"[Mesh] OR "User-Centered Design"[Mesh] OR "assistive technolog*"[tiab] OR "assistive device*"[tiab] OR "assistive product*"[tiab] OR "assistive aid*"[tiab] OR "self-help technolog*"[tiab] OR "self-help device*"[tiab] OR "self-help product*"[tiab] OR "self-help aid*"[tiab] OR "adaptive technolog*"[tiab] OR "adaptive device*"[tiab] OR "adaptive product*"[tiab] OR "adaptive aid*"[tiab] OR "User-Computer Interface"[Mesh] OR "Multimedia"[Mesh] OR "Cell phone"[Mesh] OR "Computers, handheld"[Mesh] OR "Mobile Applications"[Mesh] OR smartphone*[tiab] OR "smart phone*"[tiab] OR cellphone*[tiab] OR "cell phone*"[tiab]  OR "mobile phone*"[tiab] OR "phone application*"[tiab] OR "telephone application*"[tiab] OR "mobile application*"[tiab] OR "mobile technolog*"[tiab] OR ipad[tiab] OR ipads[tiab] OR pda[tiab] OR laptop*[tiab] OR "lap top*"[tiab] OR palmtop*[tiab] OR "palm top*"[tiab] OR "digital assistant*"[tiab] OR "interactive voice response*"[tiab] OR "voice output communication*"[tiab] OR VOCA[tiab] OR multimedia[tiab] OR multi-media[tiab] OR Mhapps[tiab] OR iphone*[tiab] OR android[tiab] OR "electronic calendar*"[tiab] OR "ball blanket*"[tiab] OR "Wearable Electronic Devices"[Mesh:NoExp] OR "Smart Glasses"[Mesh] OR (("wearable"[tiab] OR "wearables"[tiab] OR "bodyworn*"[tiab] OR "body-worn*"[tiab] OR "wristworn*"[tiab] OR "wrist-worn*"[tiab] OR "wristbased"[tiab] OR "wrist-based"[tiab] OR "earworn*"[tiab] OR "ear-worn*"[tiab]) AND ("device*"[tiab] OR "technolog*"[tiab] OR "sensor*"[tiab] OR "monitor*"[tiab])) OR "headset*"[tiab] OR "head-set*"[tiab] OR "headphone*"[tiab] OR "head-phone*"[tiab] OR "earphone*"[tiab] OR "ear-phone*"[tiab] OR "earbud*"[tiab] OR "ear-bud*"[tiab] OR "smartglass*"[tiab] OR "smart glass*"[tiab] OR "reality glass*"[tiab] OR "Google glass*"[tiab] OR "Holocue"[tiab] OR "Hololens*"[tiab] OR smartwatch*[tiab] OR "smart watch*"[tiab]** | 156,036 |

**Embase.com Session Results (20 Dec 2022)**

| Search | Query | Items found |
| --- | --- | --- |
| #6 | **#5 NOT ('conference abstract'/it OR 'conference review'/it)** | 2,844 |
| #5 | **#4 NOT (('child'/exp OR child*:ab,ti,kw OR schoolchild*:ab,ti,kw OR infant*:ab,ti,kw OR girl*:ab,ti,kw OR boy:ab,ti,kw OR boys:ab,ti,kw OR boyhood:ab,ti,kw OR youth*:ab,ti,kw OR pediatr*:ab,ti,kw OR paediatr*:ab,ti,kw OR puber*:ab,ti,kw) NOT ('adult'/exp OR 'adolescent'/exp OR 'adolescence'/exp OR adult*:ab,ti,kw OR adolescen*:ab,ti,kw OR man:ab,ti,kw OR men:ab,ti,kw OR woman:ab,ti,kw OR women:ab,ti,kw OR teen:ab,ti,kw OR teens:ab,ti,kw OR teenager*:ab,ti,kw))** | 3,889 |
| #4 | **#1 AND #2 AND #3** | 4,082 |
| #3 | **'mental disease'/exp OR 'mental patient'/exp OR 'mentally disabled person'/exp OR 'severe mental illness'/exp OR 'severe mental disorder'/exp OR 'severe mental':ab,ti,kw OR psychosis:ab,ti,kw OR psychoses:ab,ti,kw OR psychotic:ab,ti,kw OR schizo*:ab,ti,kw OR bipolar*:ab,ti,kw OR 'mental disab*':ab,ti,kw OR 'mentally disab*':ab,ti,kw OR 'psychiatric disab*':ab,ti,kw OR 'psychosocial disab*':ab,ti,kw OR 'psycho-social disab*':ab,ti,kw OR 'major depress*':ab,ti,kw OR 'anxiet*':ab,ti,kw OR 'depressive':ab,ti,kw** | 2,925,642 |
| #2 | **'quality of life'/exp OR 'daily life activity'/exp OR 'ADL disability'/exp OR 'independent living'/exp OR 'quality of life':ab,ti,kw OR 'life qualit*':ab,ti,kw OR 'living qualit*':ab,ti,kw OR 'quality of living':ab,ti,kw OR 'activities of daily living':ab,ti,kw OR 'activity of daily living':ab,ti,kw OR 'activities of daily life':ab,ti,kw OR 'activity of daily life':ab,ti,kw OR 'daily living activit*':ab,ti,kw OR 'daily life activit*':ab,ti,kw OR 'daily time':ab,ti,kw OR 'time management':ab,ti,kw OR 'adl':ab,ti,kw OR 'qol':ab,ti,kw OR 'hrql':ab,ti,kw OR 'hrqol':ab,ti,kw OR participation:ab,ti,kw OR 'social engagement':ab,ti,kw OR 'social relation*':ab,ti,kw OR 'social inclusion':ab,ti,kw OR 'social accept*':ab,ti,kw OR 'independent living*':ab,ti,kw OR 'living independent*':ab,ti,kw OR 'everyday function*':ab,ti,kw OR 'daily function*':ab,ti,kw OR 'activities of everyday':ab,ti,kw OR 'everyday liv*':ab,ti,kw OR 'everyday lif*':ab,ti,kw OR 'daily activ*':ab,ti,kw OR 'daily task*':ab,ti,kw** | 1,125,347 |
| #1 | **'self help device'/de OR 'communication aid'/exp OR 'user-centered design'/exp OR 'computer interface'/exp OR 'multimedia'/exp OR 'mobile phone'/exp OR 'personal digital assistant'/exp OR 'tablet computer'/exp OR 'laptop'/exp OR 'laptop computer'/exp OR 'mobile application'/exp OR 'mobile technology'/exp OR 'interactive voice response system'/exp OR 'assistive technology'/exp OR 'assistive technology device'/exp OR 'assistive technolog*':ab,ti,kw OR 'assistive device*':ab,ti,kw OR 'assistive product*':ab,ti,kw OR 'assistive aid*':ab,ti,kw OR 'self-help technolog*':ab,ti,kw OR 'self-help device*':ab,ti,kw OR 'self-help product*':ab,ti,kw OR 'self-help aid*':ab,ti,kw OR 'adaptive technolog*':ab,ti,kw OR 'adaptive device*':ab,ti,kw OR 'adaptive product*':ab,ti,kw OR 'adaptive aid*':ab,ti,kw OR smartphone*:ab,ti,kw OR 'smart phone*':ab,ti,kw OR cellphone*:ab,ti,kw OR 'cell phone*':ab,ti,kw  OR 'mobile phone*':ab,ti,kw OR 'phone application*':ab,ti,kw OR 'telephone application*':ab,ti,kw OR 'mobile application*':ab,ti,kw OR 'mobile technolog*':ab,ti,kw OR ipad:ab,ti,kw OR ipads:ab,ti,kw OR pda:ab,ti,kw OR laptop*:ab,ti,kw OR 'lap top*':ab,ti,kw OR palmtop*:ab,ti,kw OR 'palm top*':ab,ti,kw OR 'digital assistant*':ab,ti,kw OR 'interactive voice response*':ab,ti,kw OR 'voice output communication*':ab,ti,kw OR VOCA:ab,ti,kw OR multimedia:ab,ti,kw OR multi-media:ab,ti,kw OR Mhapps:ab,ti,kw OR iphone*:ab,ti,kw OR android:ab,ti,kw OR 'electronic calendar*':ab,ti,kw OR 'ball blanket*':ab,ti,kw OR 'wearable computer'/exp OR 'hololens'/exp OR 'smartwatch'/exp OR (('wearable':ab,ti,kw OR 'wearables':ab,ti,kw OR 'bodyworn*':ab,ti,kw OR 'body-worn*':ab,ti,kw OR 'wristworn*':ab,ti,kw OR 'wrist-worn*':ab,ti,kw OR 'wristbased':ab,ti,kw OR 'wrist-based':ab,ti,kw OR 'earworn*':ab,ti,kw OR 'ear-worn*':ab,ti,kw) AND ('device*':ab,ti,kw OR 'technolog*':ab,ti,kw OR 'sensor*':ab,ti,kw OR 'monitor*':ab,ti,kw)) OR 'headset*':ab,ti,kw OR 'head-set*':ab,ti,kw OR 'headphone*':ab,ti,kw OR 'head-phone*':ab,ti,kw OR 'earphone*':ab,ti,kw OR 'ear-phone*':ab,ti,kw OR 'earbud*':ab,ti,kw OR 'ear-bud*':ab,ti,kw OR 'smartglass*':ab,ti,kw OR 'smart glass*':ab,ti,kw OR 'reality glass*':ab,ti,kw OR 'Google glass*':ab,ti,kw OR 'Holocue':ab,ti,kw OR 'Hololens*':ab,ti,kw OR smartwatch*:ab,ti,kw OR 'smart watch*':ab,ti,kw** | 192,859 |

**APA PsycInfo (Ebsco) Session Results (20 Dec 2022)**

| Search | Query | Items found |
| --- | --- | --- |
| S5 | **S4 NOT ((ZG ("childhood (birth-12 yrs)" OR "infancy (2-23 mo)” OR "neonatal (birth-1 mo)" OR "preschool age (2-5 yrs)" OR "school age (6-12 yrs)") OR TI (child* OR schoolchild* OR infant* OR girl* OR boy* OR youth* OR pediatr* OR paediatr* OR puber*) OR AB (child* OR schoolchild* OR infant* OR girl* OR boy* OR youth* OR pediatr* OR paediatr* OR puber*)) NOT (ZG ("adulthood (18 yrs & older)" OR "adolescence (13-17 yrs)" OR "aged (65 yrs & older)" OR "middle age (40-64 yrs)" OR "thirties (30-39 yrs)" OR "very old (85 yrs & older)") OR TI (adult* OR adolescen* OR man OR men OR woman OR women OR teen OR teens OR teenager*) OR AB (adult* OR adolescen* OR man OR men OR woman OR women OR teen OR teens OR teenager*)))** | 570 |
| S4 | **S1 AND S2 AND S3** | 576 |
| S3 | **DE "Mental Disorders" OR DE "Serious Mental Illness" OR DE "Anxiety Disorders" OR DE "Bipolar Disorder" OR DE "Psychosis" OR DE "Acute Psychosis" OR DE "Affective Psychosis" OR DE "Chronic Psychosis" OR DE "Experimental Psychosis" OR DE "Reactive Psychosis" OR DE "Schizophrenia" OR DE "Affective Disorders" OR DE "Major Depression" OR DE "Schizoaffective Disorder" OR TI ("severe mental" OR psychosis OR psychoses OR psychotic OR schizo* OR bipolar* OR "mental disab*" OR "mentally disab*" OR "psychiatric disab*" OR "psychosocial disab*" OR "psycho-social disab*" OR "major depress*" OR "anxiet*" OR "depressive") OR AB ("severe mental" OR psychosis OR psychoses OR psychotic OR schizo* OR bipolar* OR "mental disab*" OR "mentally disab*" OR "psychiatric disab*" OR "psychosocial disab*" OR "psycho-social disab*" OR "major depress*" OR "anxiet*" OR "depressive") OR KW ("severe mental" OR psychosis OR psychoses OR psychotic OR schizo* OR bipolar* OR "mental disab*" OR "mentally disab*" OR "psychiatric disab*" OR "psychosocial disab*" OR "psycho-social disab*" OR "major depress*" OR "anxiet*" OR "depressive")** | 681,092 |
| S2 | **DE "Quality of Life" OR DE "Health Related Quality of Life" OR DE "Self-Care Skills" OR DE "Activities of Daily Living" OR DE "Daily Activities" OR TI ("quality of life" OR "life qualit*" OR "living qualit*" OR "quality of living" OR "activities of daily living" OR "activity of daily living" OR "activities of daily life" OR "activity of daily life" OR "daily living activit*" OR "daily life activit*" OR "daily time" OR "time management" OR "adl" OR "qol" OR "hrql" OR "hrqol" OR participation OR "social engagement" OR "social relation*" OR "social inclusion" OR "social accept*" OR "independent living*" OR "living independent*" OR "everyday function*" OR "daily function*" OR "activities of everyday" OR "everyday liv*" OR "everyday lif*" OR "daily activ*" OR "daily task*") OR AB ("quality of life" OR "life qualit*" OR "living qualit*" OR "quality of living" OR "activities of daily living" OR "activity of daily living" OR "activities of daily life" OR "activity of daily life" OR "daily living activit*" OR "daily life activit*" OR "daily time" OR "time management" OR "adl" OR "qol" OR "hrql" OR "hrqol" OR participation OR "social engagement" OR "social relation*" OR "social inclusion" OR "social accept*" OR "independent living*" OR "living independent*" OR "everyday function*" OR "daily function*" OR "activities of everyday" OR "everyday liv*" OR "everyday lif*" OR "daily activ*" OR "daily task*") OR KW ("quality of life" OR "life qualit*" OR "living qualit*" OR "quality of living" OR "activities of daily living" OR "activity of daily living" OR "activities of daily life" OR "activity of daily life" OR "daily living activit*" OR "daily life activit*" OR "daily time" OR "time management" OR "adl" OR "qol" OR "hrql" OR "hrqol" OR participation OR "social engagement" OR "social relation*" OR "social inclusion" OR "social accept*" OR "independent living*" OR "living independent*" OR "everyday function*" OR "daily function*" OR "activities of everyday" OR "everyday liv*" OR "everyday lif*" OR "daily activ*" OR "daily task*")** | 285,179 |
| S1 | **DE "Self-Help Techniques" OR DE "Assistive Technology" OR DE "Human Computer Interaction" OR DE "Multimedia" OR DE "Mobile Devices" OR DE "Mobile Phones" OR DE "Smartphones" OR DE "Tablet Computers" OR DE "Mobile Technology" OR DE "Mobile Applications" OR DE "Laptop Computers" OR DE "Wearable Devices" OR TI ("assistive technolog*" OR "assistive device*" OR "assistive product*" OR "assistive aid*" OR "self-help technolog*" OR "self-help device*" OR "self-help product*" OR "self-help aid*" OR "adaptive technolog*" OR "adaptive device*" OR "adaptive product*" OR "adaptive aid*" OR smartphone* OR "smart phone*" OR cellphone* OR "cell phone*"  OR "mobile phone*" OR "phone application*" OR "telephone application*" OR "mobile application*" OR "mobile technolog*" OR ipad OR ipads OR pda OR laptop* OR "lap top*" OR palmtop* OR "palm top*" OR "digital assistant*" OR "interactive voice response*" OR "voice output communication*" OR VOCA OR multimedia OR multi-media OR Mhapps OR iphone* OR android OR "electronic calendar*" OR "ball blanket*" OR (("wearable" OR "wearables" OR "bodyworn*" OR "body-worn*" OR "wristworn*" OR "wrist-worn*" OR "wristbased" OR "wrist-based" OR "earworn*" OR "ear-worn*") AND ("device*" OR "technolog*" OR "sensor*" OR "monitor*")) OR "headset*" OR "head-set*" OR "headphone*" OR "head-phone*" OR "earphone*" OR "ear-phone*" OR "earbud*" OR "ear-bud*" OR "smartglass*" OR "smart glass*" OR "reality glass*" OR "Google glass*" OR "Holocue" OR "Hololens*" OR smartwatch* OR "smart watch*") OR AB ("assistive technolog*" OR "assistive device*" OR "assistive product*" OR "assistive aid*" OR "self-help technolog*" OR "self-help device*" OR "self-help product*" OR "self-help aid*" OR "adaptive technolog*" OR "adaptive device*" OR "adaptive product*" OR "adaptive aid*" OR smartphone* OR "smart phone*" OR cellphone* OR "cell phone*"  OR "mobile phone*" OR "phone application*" OR "telephone application*" OR "mobile application*" OR "mobile technolog*" OR ipad OR ipads OR pda OR laptop* OR "lap top*" OR palmtop* OR "palm top*" OR "digital assistant*" OR "interactive voice response*" OR "voice output communication*" OR VOCA OR multimedia OR multi-media OR Mhapps OR iphone* OR android OR "electronic calendar*" OR "ball blanket*" OR (("wearable" OR "wearables" OR "bodyworn*" OR "body-worn*" OR "wristworn*" OR "wrist-worn*" OR "wristbased" OR "wrist-based" OR "earworn*" OR "ear-worn*") AND ("device*" OR "technolog*" OR "sensor*" OR "monitor*")) OR "headset*" OR "head-set*" OR "headphone*" OR "head-phone*" OR "earphone*" OR "ear-phone*" OR "earbud*" OR "ear-bud*" OR "smartglass*" OR "smart glass*" OR "reality glass*" OR "Google glass*" OR "Holocue" OR "Hololens*" OR smartwatch* OR "smart watch*") OR KW ("assistive technolog*" OR "assistive device*" OR "assistive product*" OR "assistive aid*" OR "self-help technolog*" OR "self-help device*" OR "self-help product*" OR "self-help aid*" OR "adaptive technolog*" OR "adaptive device*" OR "adaptive product*" OR "adaptive aid*" OR smartphone* OR "smart phone*" OR cellphone* OR "cell phone*"  OR "mobile phone*" OR "phone application*" OR "telephone application*" OR "mobile application*" OR "mobile technolog*" OR ipad OR ipads OR pda OR laptop* OR "lap top*" OR palmtop* OR "palm top*" OR "digital assistant*" OR "interactive voice response*" OR "voice output communication*" OR VOCA OR multimedia OR multi-media OR Mhapps OR iphone* OR android OR "electronic calendar*" OR "ball blanket*" OR (("wearable" OR "wearables" OR "bodyworn*" OR "body-worn*" OR "wristworn*" OR "wrist-worn*" OR "wristbased" OR "wrist-based" OR "earworn*" OR "ear-worn*") AND ("device*" OR "technolog*" OR "sensor*" OR "monitor*")) OR "headset*" OR "head-set*" OR "headphone*" OR "head-phone*" OR "earphone*" OR "ear-phone*" OR "earbud*" OR "ear-bud*" OR "smartglass*" OR "smart glass*" OR "reality glass*" OR "Google glass*" OR "Holocue" OR "Hololens*" OR smartwatch* OR "smart watch*")** | 52,155 |

**Web of Science (Core Collection) Session Results (20 Dec 2022)**

| Search | Query | Items found |
| --- | --- | --- |
| #5 | **#4 NOT TS=((child* OR schoolchild* OR infant* OR girl* OR boy OR boys OR boyhood OR youth* OR pediatr* OR paediatr* OR puber*) NOT (adult* OR adolescen* OR man OR men OR woman OR women OR teen OR teens OR teenager*))** | 931 |
| #4 | **#1 AND #2 AND #3** | 972 |
| #3 | **TS=("severe mental" OR psychosis OR psychoses OR psychotic OR schizo* OR bipolar* OR "mental disab*" OR "mentally disab*" OR "psychiatric disab*" OR "psychosocial disab*" OR "psycho-social disab*" OR "major depress*" OR "anxiet*" OR "depressive")** | 918,161 |
| #2 | **TS=("quality of life" OR "life qualit*" OR "living qualit*" OR "quality of living" OR "activities of daily living" OR "activity of daily living" OR "activities of daily life" OR "activity of daily life" OR "daily living activit*" OR "daily life activit*" OR "daily time" OR "time management" OR "adl" OR "qol" OR "hrql" OR "hrqol" OR participation OR "social engagement" OR "social relation*" OR "social inclusion" OR "social accept*" OR "independent living*" OR "living independent*" OR "everyday function*" OR "daily function*" OR "activities of everyday" OR "everyday liv*" OR "everyday lif*" OR "daily activ*" OR "daily task*")** | 1,036,960 |
| #1 | **TS=("assistive technolog*" OR "assistive device*" OR "assistive product*" OR "assistive aid*" OR "self-help technolog*" OR "self-help device*" OR "self-help product*" OR "self-help aid*" OR "adaptive technolog*" OR "adaptive device*" OR "adaptive product*" OR "adaptive aid*" OR smartphone* OR "smart phone*" OR cellphone* OR "cell phone*"  OR "mobile phone*" OR "phone application*" OR "telephone application*" OR "mobile application*" OR "mobile technolog*" OR ipad OR ipads OR pda OR laptop* OR "lap top*" OR palmtop* OR "palm top*" OR "digital assistant*" OR "interactive voice response*" OR "voice output communication*" OR VOCA OR multimedia OR multi-media OR Mhapps OR iphone* OR android OR "electronic calendar*" OR "ball blanket*" OR (("wearable" OR "wearables" OR "bodyworn*" OR "body-worn*" OR "wristworn*" OR "wrist-worn*" OR "wristbased" OR "wrist-based" OR "earworn*" OR "ear-worn*") AND ("device*" OR "technolog*" OR "sensor*" OR "monitor*")) OR "headset*" OR "head-set*" OR "headphone*" OR "head-phone*" OR "earphone*" OR "ear-phone*" OR "earbud*" OR "ear-bud*" OR "smartglass*" OR "smart glass*" OR "reality glass*" OR "Google glass*" OR "Holocue" OR "Hololens*" OR smartwatch* OR "smart watch*")** | 232,397 |
