# Supplementary material for: Use of Assistive Technology for Persons with Psychosocial Disability: Systematic Review
Source: JMIR Rehabil Assist Technol. 2023 Nov 15;10:e49750. doi: 10.2196/49750 (PMC10687692; doi:10.2196/49750)
Supplement: Multimedia Appendix 2 [file rehab_v10i1e49750_app2.docx]

**Table S1.** Study characteristics, objectives, and assistive product use and outcomes.

| Lead author, year | Study design, n* | Country | Objective | Psychosocial disability | AP^a^ and pattern of use | Outcomes | MMAT^b^ quality assessment |
| --- | --- | --- | --- | --- | --- | --- | --- |
| Kimhy and Corcoran [23], 2008 | Qualitative—case report; n=1 | United States | To assess the feasibility and effectiveness of a palm computer to improve homework completion and overcome treatment barriers associated with negative symptoms. | Schizophrenia | Palm tungsten T3 handheld computer: User completion of momentary logs of mood, thoughts, behaviour, and social context. | Increase in productivity for completing schoolwork. Reductions in feelings of anxiety, guilt, and depression. Fewer difficulties in expressing thoughts and increased ability to recognize dysfunctional thoughts. | 3/4 moderate-strong |
| Sablier et al [24], 2012 | Quantitative— experimental study; n=9 | France | To explore the use and satisfaction of MOBUS by people with schizophrenia. | Schizophrenia | MOBUS, PDA^c^ supplies cognitive assistance and telemonitoring for ADLs^d^, as well as tools to gather ecological medical data through 2 connected subapplications implemented in PDA: one for the patients, the other for the caregivers. | Three instances of increased ambition to try new activities and increase social function. | 3/4 moderate-strong |
| Sajatovic et al [25], 2015 | Quantitative—prospective study; n=5 | United States | To evaluate preliminary feasibility, patient satisfaction and effects on adherence, bipolar disorder knowledge, and bipolar disorder symptoms associated with the use of a multicomponent technology-assisted adherence enhancement system. | Bipolar disorder | Automated pill cap; MAE^e^ and TIP^f^. The automated pill cap with remote monitoring sensor—sensor records the instance of the bottle opening and stores it in memory, tracking patients’ presumed dosing episodes; MAE educated patients about their illness and self-management skills for a successful clinical outcomes program; and TIP motivates patients to remain adherent and improve treatment-related knowledge and skills. | Improved adherence to medication and treatment. | 4/4 strong |
| Ekholm et al [26], 2020 | Quantitative—randomized control trial; n=120 | Sweden | To evaluate the effect of weighted chain blankets on insomnia and sleep-related daytime symptoms for patients with major depressive disorder, bipolar disorder, generalized anxiety disorder, and attention-deficit/hyperactivity disorder. | Major depressive disorder, bipolar disorder and generalized anxiety disorder | Weighted chain blankets apply pressure to the whole body for deep pressure stimulation to target sleep issues and calm anxiety symptoms. | Increased sleep maintenance, higher daytime activity level, and reduced daytime symptoms of fatigue, anxiety, and depression. | 4/4 strong |
| Resta et al [27], 2021 | Quantitative—experimental study, nonconcurrent multiple baseline design; n=14 | Italy | To assess a smartphone-based system to remind the user to initiate functional daily activities (eg, setting a table for lunch) and perform them without the support of a caregiver. | Schizophrenia and depression | Smartphone function (nonspecialty app) fitted with the Easy Alarm YouTube app, alerts sent to participants at a time to start daily scheduled activities and instructions for the steps of that activity. | Significant increase in the number of activity steps correctly performed when supported by the prompting. | 3/4 moderate-strong |

^a^AP: assistive product.

^b^MMAT: Mixed Methods Appraisal Tool.

^c^PDA: personal digital assistant.

^d^ADL: activity of daily living.

^e^MAE: multimedia adherence enhancement.

^f^TIP: treatment incentive program.
