# Supplementary material for: Use of Assistive Technology for Persons with Psychosocial Disability: Systematic Review
Source: JMIR Rehabil Assist Technol. 2023 Nov 15;10:e49750. doi: 10.2196/49750 (PMC10687692; doi:10.2196/49750)
Supplement: Multimedia Appendix 3 [file rehab_v10i1e49750_app3.docx]

**Multimedia Appendix 3.** Assistive technology for psychosocial disability.

| **Study** | **Assistive Technology** | **Psychosocial disability** | **Sample Image of Assistive Technology** | **Benefits** |
| --- | --- | --- | --- | --- |
| Kimhy & Corcoran, 2008 [23] | Palm Tungsten T3 handheld computer: | Schizophrenia | 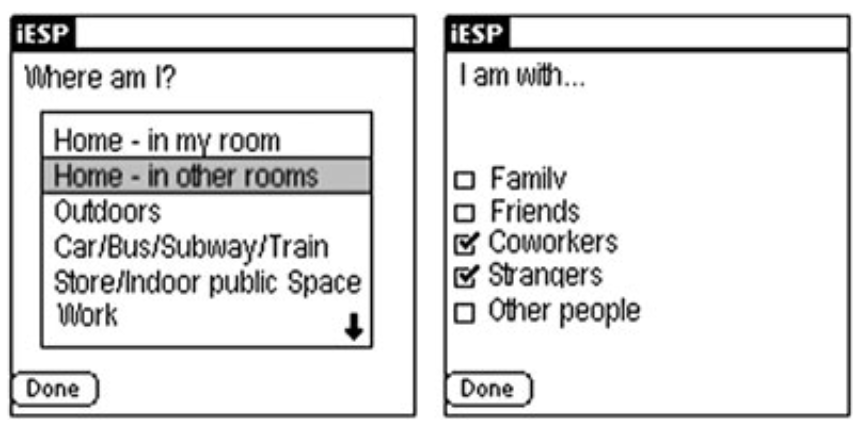  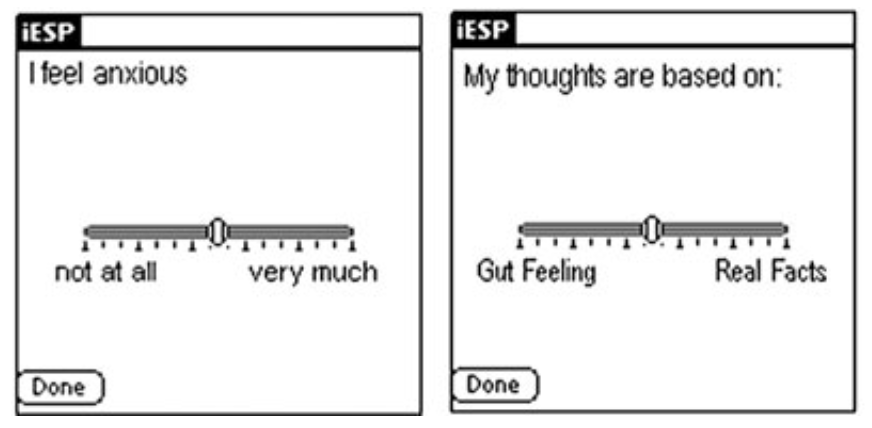 | Improves task completion and independent initiation. |
| Sablier et al., 2012  [24] | MOBUS, personal digital assistant (PDA) | Schizophrenia | 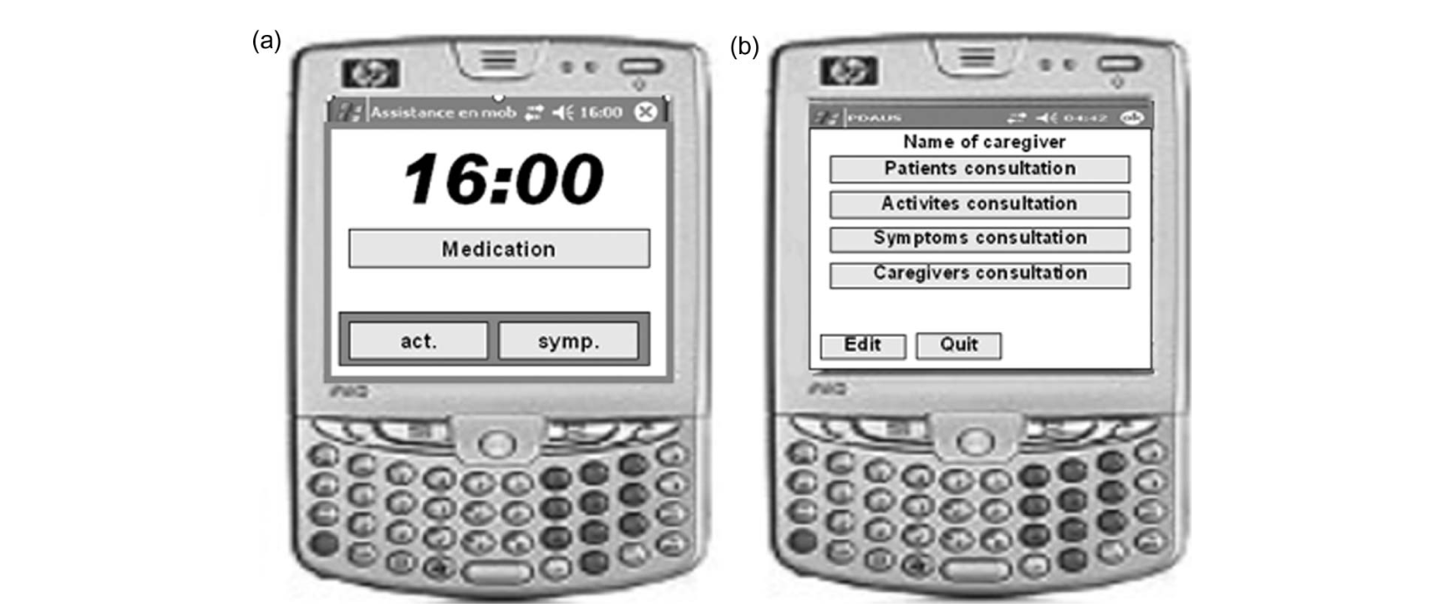 | Increases ambition to try new activities and increase social function. |
| Sajatovic et al., 2015 [25] | Automated pill cap | Bipolar Disorder | 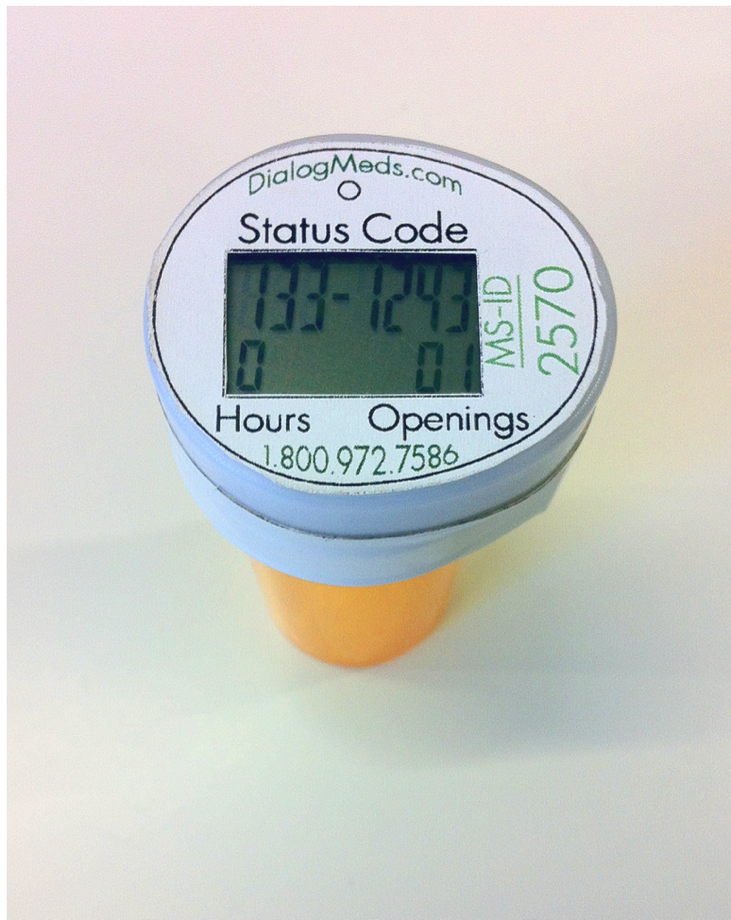 | Improves adherence to medication and treatment |
| Ekholm et al.,2020 [26]* | Weighted chain blankets | Major  depressive disorder, Bipolar disorder and generalized anxiety disorder | 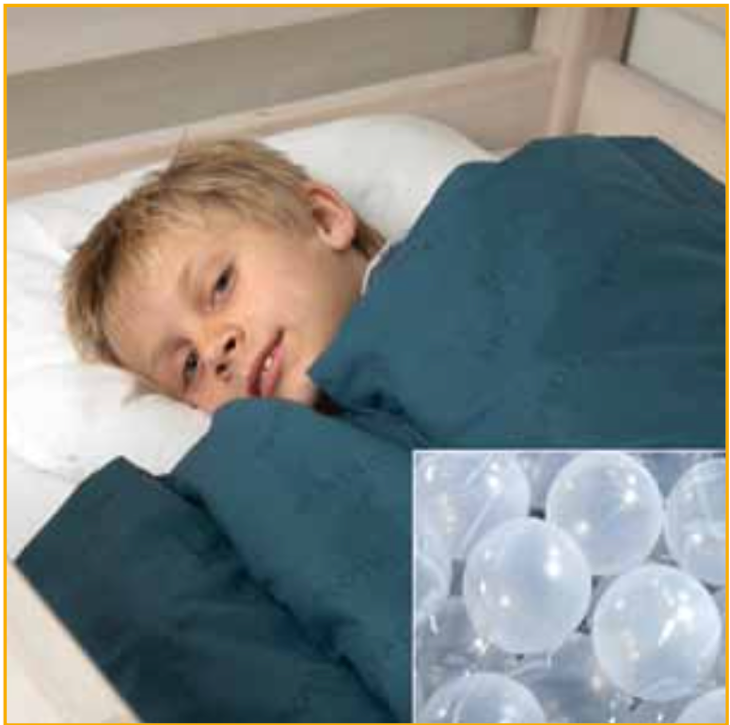 | Improves symptoms of anxiety by creating a “calming” effect on the body |
| Resta et al.,2021 [27]^+^ | Smartphone function (non-specialty application) | Schizophrenia and Depression | 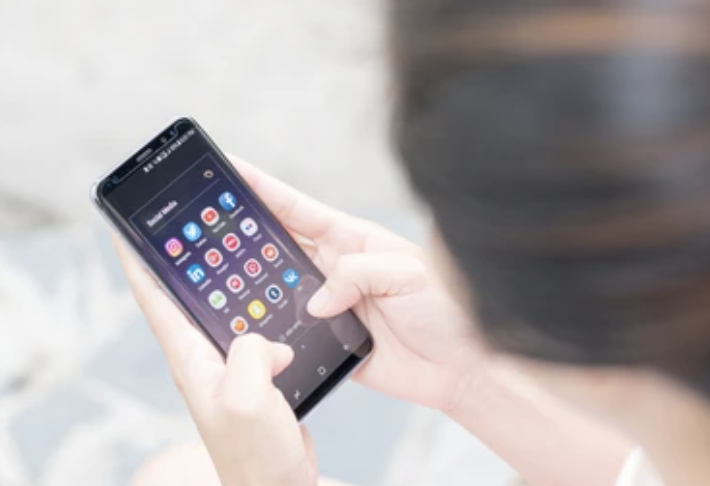 | Improves number of activities initiated independently and number of tasks completed successfully. |

*Image is of a weighted ball blanket, taken adapted from Wallskr & Jensen, 2012 [8] as the article (Ekholm et al.,2020) in the manuscript did not include an image of the specific assistive technology

^+^Image is a stock image adapted from Shutterstock.com, (Stock Photo ID: 662197531) 2017; as the article (Resta et al.,2021) in the manuscript did not include an image of the specific assistive technology.
